# Complications on the feeding artery after an arterio-venous fistula closure in patients after kidney transplantation – a national cohort study

**DOI:** 10.1093/ckj/sfae360

**Published:** 2024-11-15

**Authors:** Matej Zrimšek, Barbara Vajdič-Trampuž, Matija Jelenc, Juš Kšela, Jakob Gubenšek

**Affiliations:** Faculty of Medicine, University of Ljubljana, Ljubljana, Slovenia; Department of Nephrology, University Medical Centre Ljubljana, Ljubljana, Slovenia; Faculty of Medicine, University of Ljubljana, Ljubljana, Slovenia; Department of Nephrology, University Medical Centre Ljubljana, Ljubljana, Slovenia; Faculty of Medicine, University of Ljubljana, Ljubljana, Slovenia; Department of Cardiovascular Surgery, University Medical Centre Ljubljana, Ljubljana, Slovenia; Faculty of Medicine, University of Ljubljana, Ljubljana, Slovenia; Department of Cardiovascular Surgery, University Medical Centre Ljubljana, Ljubljana, Slovenia; Faculty of Medicine, University of Ljubljana, Ljubljana, Slovenia; Department of Nephrology, University Medical Centre Ljubljana, Ljubljana, Slovenia

**Keywords:** arterial thrombosis, arteriovenous fistula, complications, true brachial artery aneurysm, ultrasound exam

## Abstract

**Background:**

Arteriovenous fistulas (AVFs) in kidney transplant recipients are sometimes closed, either as a policy or due to complications. We collected data on the incidence of complications after AVF closure in a national cohort of transplanted patients.

**Methods:**

Patients who received a kidney transplant between 2000 and 2015 and had a functional AVF that was later ligated or extirpated were included. Medical records were searched for arterial complications on the arm with the closed AVF. Furthermore, all patients who were still alive in January 2023 were invited for a follow-up arterial ultrasound exam.

**Results:**

Sixty patients were included; mean follow-up was 9.3 ± 3.8 years. There were five (8% cumulative incidence) patients with symptomatic arterial thrombosis and three (5% incidence) with a symptomatic feeding artery aneurysm. Prospective ultrasound exams were performed in 50 patients; the mean diameter of the brachial artery was almost doubled on the arm with the closed AVF (8.1 ± 3.2 versus 4.7 ± 0.7 mm; *P* < .001). Additional asymptomatic complications were found in nine patients (18% incidence): seven cases (14% incidence) of arterial thrombosis, some extending up to the axillary artery, and three (6% incidence) brachial artery aneurysms. All patients in whom the thrombosis spread to the brachial artery had large brachial arteries (>10 mm) or an aneurysm.

**Conclusion:**

We observed a high cumulative incidence of arterial thrombosis (20%) and brachial artery aneurysms (10%), sometimes developing several years after AVF closure. These complications should be taken into account when contemplating closure of a well-developed AVF and an AVF-preserving approach with flow reduction surgery might be preferred in some cases.

## Graphical Abstract

**Graphical Abstract:**
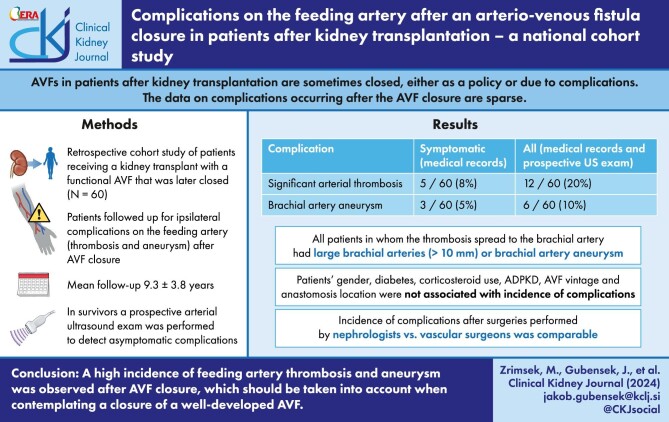

KEY LEARNING POINTS **What was known:**

- There is no universally accepted strategy for either preservation or ligation of an AVF after successful kidney transplantation. AVFs are sometimes closed in patients with well-functioning kidney transplants, either as a policy, at the patient's request or due to complications.
- Despite the possibility that routine AVF closure after kidney transplantation may reduce the risk of AVF complications and have a positive impact on cardiac function, a functioning AVF can sometimes be of use and AVF closure with resulting thrombosis or extirpation of the fistula vein often reduces future options for vascular access.
- The data in the literature on complications occurring after closure of an AVF are sparse. Aneurysms of the feeding artery are described as one of the complications and may be associated with thrombosis and embolism. The incidence of complications, which would inform decision-making, is not well established.

**This study adds:**

- Among 60 kidney transplant patients after AVF closure, 8% of patients had symptomatic arterial thrombosis and 5% had a symptomatic feeding artery aneurysm.
- Prospective ultrasound revealed additional asymptomatic arterial complications in nine patients (18% incidence). The incidence of asymptomatic arterial thrombosis was 14% and some extended up to the axillary artery. The incidence of brachial artery aneurysms was 6%.
- The incidence of complications after surgeries performed by nephrologists versus vascular surgeons was comparable.

**Potential impact:**

- Feeding artery complications (thrombosis and aneurysms) sometimes develop several years after AVF closure and are much more common than previously thought. They should be considered when contemplating closure of a well-developed AVF and an AVF-preserving approach with flow reduction surgery might be preferred in some cases.

## INTRODUCTION

The optimal management of a functioning arteriovenous fistula (AVF) in kidney transplant recipients remains controversial and there is no universally accepted strategy for either preservation or ligation of the AVF after a successful transplantation. AVF closure is sometimes performed in patients with well-functioning kidney transplants, either as a policy, at the patient's request or because of complications. In the most recent guidelines of the European Society for Vascular Surgery [1], routine closure of a functioning vascular access after successful kidney transplantation is not recommended but should be considered in patients with refractory heart failure.

Balancing the potential benefits of vascular access ligation (improvement in kidney function, reversal of adverse effects on the heart) and its preservation (maintenance of access for the time of return to dialysis) against potential complications of vascular access (such as aneurysms, development of high flow) in each individual patient remains a challenge. In our recent national cohort study, we reported that the AVF remained functional in the majority of our patients after kidney transplantation (centre policy) and that complications related to the AVF were common [2]. The most frequent were aneurysmal enlargement of the fistula vein and painful thrombosis with associated thrombophlebitis that required appropriate treatment [2]. Despite the possibility that routine AVF closure after kidney transplantation may reduce the risk of AVF complications and have a beneficial effect on cardiac function [3–6], it is important to recognize that a functioning AVF can sometimes be of use and that AVF closure with resultant thrombosis or extirpation of the fistula vein often results in a reduction of ‘venous capital’, which reduces future options for vascular access and could, in some cases, reduce patients’ quality of life or even life span. Wilmink *et al.* [7] have recently proposed guidelines for AVF closure after kidney transplantation, basing the decision on a trade-off between the estimated probability of future graft failure and the probability of future damage from a well-functioning fistula. We believe that complications occurring after vascular access closure should also be taken into consideration.

The data in the literature on complications occurring after closure (ligation or extirpation) of an AVF are sparse. In case of simple ligation of the fistula vein without artery reconstruction, the development of aneurysms from the stump of the fistula vein (including the anastomotic area) has been described [8]. In rare cases, thrombosis of the feeding artery can occur immediately after ligation or extirpation [6, 9]. Aneurysms of the feeding artery, unrelated to the site of anastomosis, are also described as a late complication. They can develop in functioning high-flow AVFs [10–12], but occur more frequently after AVF ligation, most often in transplant patients on corticosteroids [10–15]. A true aneurysm of the feeding artery may be associated with thrombosis and embolism resulting in distal limb ischaemia, nerve compression and, very rarely, aneurysm rupture [13, 16–18]. The incidence of arterial complications, which would inform decision-making, is not well established, as mostly series of patients who required surgical treatment are reported [15]. There are very few cross-sectional or prospective follow-up cohorts reported in the literature [11, 18], which would enable an estimation of the incidence of this complication.

The aim of our study was to report the incidence of complications after AVF closure in a national cohort of kidney transplant recipients.

## MATERIALS AND METHODS

### Study design

The study was based on a previously published national cohort of kidney transplant recipients from Slovenia who were transplanted between 1 January 2000 and 31 December 2015 and had a functioning AVF/graft at the time of transplantation [2]. In the present study, only patients who had their functioning AVF closed (ligated or extirpated) after receiving kidney graft were included. The start of the observation period was at the time of AVF closure and patients were followed up for complications until January 2023 or death. We excluded patients whose medical records were not available and patients who had another vascular access placed on the same arm. Electronic medical records were searched for symptomatic complications that occurred in the arterial system of the arm with a closed AVF for which patients sought medical attention. Furthermore, patients alive in January 2023 were invited for an ultrasound exam of the arteries of the arm with the closed AVF to detect any clinically asymptomatic complications of the arterial system.

The study was approved by the National Medical Ethics Committee (ref. no. 0120-483/2022/3). The consent was waived for the retrospective part of the study, which was based on existing medical records, while patients gave written informed consent for the prospective ultrasound exam.

### Retrospective data collection

Patients’ medical records were reviewed and data on the time since kidney transplantation, comorbid conditions, site of a functional AVF, time from AVF construction to its closure, reasons for AVF closure, who performed the surgery [nephrologist with surgical skills or (cardio)vascular surgeon] and serum creatinine at the time of AVF closure were collected. Nephrologists with surgical skills mainly performed a simple fistula vein ligation close to the AV anastomosis (resulting in a fistula vein stump) under local anaesthesia; in rare cases, a small aneurysm of the fistula vein was also resected. (Cardio)vascular surgeons, using general anaesthesia, usually removed a significant portion of the large/aneurysmatic fistula vein (extirpation) and most often reconstructed the feeding artery (i.e. leaving no fistula vein stump). Medical records up to January 2023 were screened for complications associated with the arm with the closed AVF. These complications are reported as symptomatic, as the patients sought medical attention. Immediate postoperative surgical complications were not the focus of the present study and are therefore not reported, while complications occurring at least a few weeks after surgery are reported.

### Prospective screening ultrasound exam

Patients who were still alive in January 2023 were invited for a screening ultrasound exam of the arm with the closed AVF; the response rate was 100%. During the ultrasound exam, the arterial system of the arm with the closed AVF was examined from the axilla to the site of the former AVF anastomosis for new significant findings. The presence of aneurysms, arterial kinking, complete thrombosis or the presence of mural thrombi within the arterial system was recorded. These complications are reported as asymptomatic, as they were discovered solely due to the ultrasound exam performed for study purposes. Although some of the patients did have some mild symptoms when asked directly, they did not seek medical attention.

An arterial aneurysm was defined as doubling of the diameter of the artery compared with the more proximal part or an absolute diameter ≥2 cm for the brachial artery or ≥1.5 cm for the radial/ulnar artery. An aneurysmatic dilatation (usually with partial thrombosis) of the anastomotic area and the initial part of the fistula vein stump (after simple AVF ligation) was not considered an arterial aneurysm and was recorded separately. Arterial kinking was defined as >90° curvature of the artery and was diagnosed when another lumen of the artery was seen on a single ultrasound image while the original lumen was viewed cross-sectionally. Complete thrombosis was defined by the presence of intraluminal thrombi and non-compressibility of the lumen. Thrombosis involving only a short perianastomotic segment of the radial artery, which is a relatively common finding, was not counted as significant arterial thrombosis and was reported separately. In patients without detected complications, the diameter of the widest part of the brachial artery on the ipsi- and contralateral arm (if an AVF was never constructed in the contralateral arm) was recorded. If a complication was detected, patients were treated with acetylsalicylic acid or anticoagulants or were considered for surgical intervention. The data on their treatment are also presented.

### Statistical analysis

Descriptive statistics were used and data were summarized as frequencies (percentages), means and standard deviations (SDs) or medians and interquartile ranges (IQRs) for non-normally distributed data. The incidence of complications was presented separately for symptomatic and asymptomatic complications and cumulative data on the incidence of all complications were also reported. Groups were compared with paired *t*-test or Fisher's exact test, as appropriate. A *P*-value <.05 was considered statistically significant.

## RESULTS

**Figure 1:**
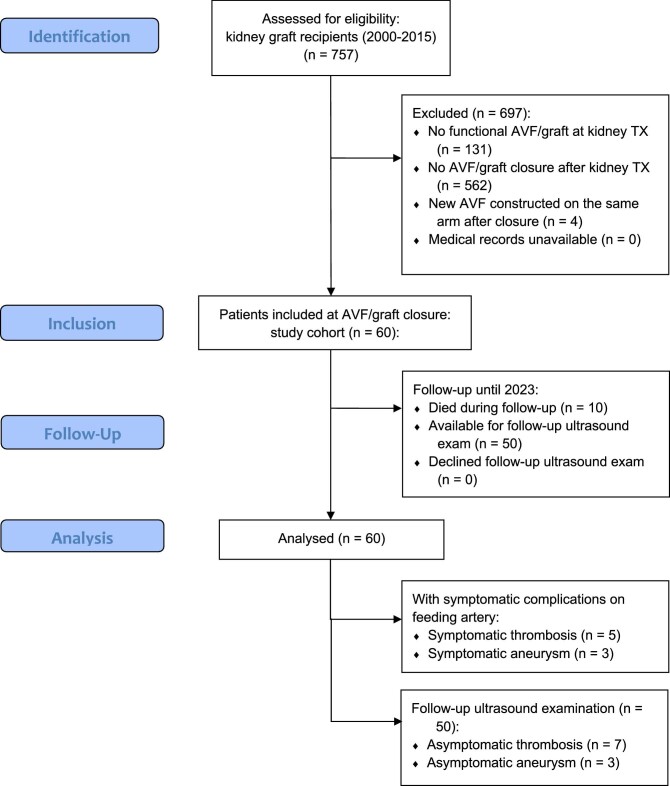
Flow chart of patient selection.

This study was based on a previously published cohort of 757 adult kidney transplant patients from 2000 to 2015, of whom 626 had a functional AVF/graft at the time of transplantation [2]. In the present study, 60 patients who had their AVF closed (ligated or extirpated) after receiving a kidney transplant were included (see Fig. 1). The characteristics of the patients and their AVFs are shown in Table 1. Patients were followed for a mean of 9.3 ± 3.8 years after AVF closure.

**Table 1:** Characteristics of patients and their AVFs at the time of inclusion in the study (i.e. at AVF closure).

Characteristics	Values
Patients, *N*	60
Age (years), mean ± SD	54 ± 11
Female, *n* (%)	26 (43)
Hypertension, *n* (%)	42 (70)
Diabetes, *n* (%)	16 (27)
Autosomal dominant polycystic kidney disease, *n* (%)	9 (15)
Serum creatinine (μmol/l), mean ± SD	100 ± 28
Time since kidney transplant (years), median (IQR)	4 (2–6)
Anticoagulant therapy at time of AVF closure, *n* (%)	2 (3)
Corticosteroids at time of AVF closure, *n* (%)	37 (62)
AVF anastomosis location, *n* (%)	
Forearm	36 (60)
Elbow	24 (40)
Time from AVF formation to closure (years), median (IQR)	8 (5–11)
Reason for AVF closure, *n* (%)	
Aneurysmatic/large/high-flow AVF	43 (72)
Distal ischaemia	6 (10)
Patient's preference	6 (10)
Partial thrombosis with thrombophlebitis	2 (3)
Risk of rupture	1 (2)
Other/unknown	2 (3)
Surgery performed by, *n* (%)	
Nephrologist with surgical skills	28 (47)
(Cardio)vascular surgeon	32 (53)

Retrospective review of medical records revealed that seven patients had symptoms in the arm with the closed AVF and sought medical attention, on average 4 ± 2 years after AVF closure. Some patients had more than one type of complication occurring concomitantly. Their cases are summarized in Table 2. There were five patients (8% cumulative incidence) with symptomatic arterial thrombosis after AVF closure. All of them had either a high-flow or aneurysmatic AVF with a brachial artery diameter >10 mm. Patients with pain at rest (*n* = 2) were treated with local thrombolysis or surgical thrombectomy, those without pain at rest (*n* = 2) received anticoagulant therapy and one patient with asymptomatic radial artery thrombosis (with a concomitant symptomatic brachial artery aneurysm) continued to receive acetylsalicylic acid. Clinical outcomes were excellent (see Table 2). In the short term, there was no necrosis or amputation and in the long term, patients were able to use their arms without symptoms or were only unable to perform heavier tasks. Furthermore, there were three patients (5% cumulative incidence) with a symptomatic, complicated (i.e. with mural thrombus) aneurysm of the feeding artery (see Table 2). Two patients were treated with aneurysm resection and artery reconstruction and one was placed on anticoagulant therapy. Clinical outcomes were excellent and none of the patients had further complications. Separately, four patients had partially thrombosed aneurysms of the initial stump of the fistula vein, involving also the anastomotic area, which were relatively large and were surgically resected with artery reconstruction.

**Table 2:** Summary of symptomatic complications after AVF closure (retrospective data collection).

Patient, age (years) and gender	AVF type and vintage (at closure)	Reasons for AVF closure	Antiplatelet and anticoagulant/CS therapy (at closure)	Time since AVF closure and type of feeding artery complication	Complication details	Treatment	Long-term clinical outcome
case 1, 46, male	RCAVF, 7 years	Aneurysms	No/CS+	5 years, thrombosis	Thrombosis of RA from anastomosis to bifurcation, progressing to axilla during the next 8 years despite anticoagulant therapy; BA diameter 1.6 cm	Anticoagulant therapy	Cannot perform heavier tasks with the affected arm
case 2, 71, male	RCAVF, 6 years	High flow	ASA/CS−	4 years, thrombosis	Arterial thrombosis from anastomosis up to axillary artery; BA diameter 1.3 cm	Iloprost infusion, anticoagulant therapy	No task limitation with the affected arm
case 3, 61, male	RCAVF, 4 years	Aneurysms	ASA/CS+	1 year, thrombosis	Arterial thrombosis from anastomosis up to axillary artery; BA diameter 1.2 cm	Thrombolysis—unsuccessful, surgical thrombectomy	No task limitation with the affected arm
case 4, 54, male	BCAVF, 11 years	High-flow	ASA/CS+	1 year, thrombosis	Kinking of BA and mural thrombus, progressing to full thrombosis from midarm to anastomosis; BA diameter 1.4 cm	Surgical thrombectomy, anticoagulant therapy	No task limitation with the affected arm
case 5, 74, female	RCAVF, 10 years	Aneurysms	ASA/CS−	5 years, aneurysm and thrombosis	BA aneurysm (1.5 cm diameter) with mural thrombus and RA thrombosis from anastomosis to bifurcation	Aneurysm resection, continued on ASA	Cannot perform heavier tasks with the affected arm
case 6, 68, male	RCAVF, 10 years	Aneurysms and high flow	No/CS−	5 years, aneurysm	BA aneurysm (5 cm diameter) with mural thrombus	Aneurism resection and graft interposition	No task limitation with the affected arm
case 7, 61, male	BCAVF, 5 years	Aneurysms	No/CS−	7 years, aneurysm	BA aneurysm (2.8 cm diameter) with mural thrombus	Anticoagulant therapy	No task limitation with the affected arm

ASA: acetylsalicylic acid 100 mg/day; BA: brachial artery; BCAVF: brachiocephalic arteriovenous fistula; CS: corticosteroids; RA: radial artery; RCAVF: radiocephalic arteriovenous fistula.

During the follow-up period, 10 patients died; the majority (8/10) died due to infection, 1 death was cardiovascular and 1 due to malignancy. In the remaining 50 patients, a prospective ultrasound exam was performed. The mean diameter of the brachial artery on the ipsilateral side of the closed AVF (excluding patients with aneurysm or thrombosis) was 8.1 ± 3.2 mm, while on the contralateral side it was 4.7 ± 0.7 mm (*P* < .001). Additional relevant arterial complications were found in nine patients (18% cumulative incidence), which are summarized in Table 3 and the incidence is calculated in Table 4. Among them, there were seven cases (14% cumulative incidence) of arterial thrombosis found. About half of them affected the whole radial artery, while others extended higher, some up to the axillary artery. As with symptomatic patients, all patients with arterial thrombosis extending to the brachial artery had large brachial arteries (>10 mm) or an aneurysm of the brachial artery. There were three cases (6% cumulative incidence) of brachial artery aneurysm found, one of which required surgical treatment and one of which was associated with thrombosis that spread to the anastomosis on the radial artery. Among less severe complications, there were two thromboses of a short perianastomotic segment of the radial artery and nine (18% cumulative incidence) kinked segments of the brachial artery. Because a complete ultrasound exam was often not performed before AVF closure, we do not know whether the kinking occurred before (in high-flow AVF) or after AVF closure.

**Table 3:** Summary of significant arterial complications in the arm with a closed AVF found in asymptomatic patients on prospective ultrasound examination.

Patient, age (years) and gender	AVF type and vintage (at closure)	Reasons for and type of AVF closure	Surgical complications at AVF closure^a^	Antiplatelet and anticoagulant/CS therapy (at closure)	Time since AVF closure and type of feeding artery complication	Complication details	Treatment
case 8, 43, male	RCAVF, 7 years	Patient preference	No	ASA/CS+	13 years, thrombosis	Arterial thrombosis from anastomosis to RA branching	None
case 9, 63, male	RCAVF, 17 years	Large AVF	No	No/CS−	7 years, thrombosis	Arterial thrombosis from anastomosis to RA branching	ASA
case 10, 51, male	RCAVF, 17 years	Aneurysms	No	No/CS+	10 years, aneurysm and thrombosis	Thrombosis from anastomosis on RA to mid-BA + BA aneurysm (3.1 cm diameter)	ASA
case 11, 56, female	BCAVF, 5 years	Venous hypertension	Thrombophlebitis	No/CS+	14 years, thrombosis	Arterial thrombosis from anastomosis to axillary artery (BA diameter 1.8 cm)	Already on anticoagulant therapy for other indication
case 12, 54, female	BCAVF, 4 years	High flow	No	No/CS+	11 years, thrombosis	Arterial thrombosis from anastomosis to axillary artery (BA diameter 1.2 cm)	ASA
case 13, 75, female	RCAVF, 18 years	Aneurysms	Thrombophlebitis	No/CS−	10 years, thrombosis	Arterial thrombosis from anastomosis to RA branching	None, anticoagulant therapy for other indication
case 14, 52, male	BBAVF, 5 years	Aneurysms	No	Anticoagulant therapy (post-DVT), CS+	11 years, aneurysm	Small (1.1 cm) BA aneurysm on upper arm	None, anticoagulant therapy for other indication
case 15, 70, male	BCAVF, 3 years	Large AVF	No, several revisions at another surgery^b^	ASA, CS+	11 years, thrombosis	Thrombosis of perianastomotic segment of BA	Already taking ASA
case 3, 68, male	RCAVF, 4 years	Aneurysms	No	ASA, CS+	8 years, aneurysm	BA aneurysm (4.4 cm) with mural thrombus	Aneurysm resection with graft interposition planned

ASA; acetylsalicylic acid 100 mg/day; BA: brachial artery; BBAVF: brachiobasilic arteriovenous fistula; BCAVF: brachiocephalic arteriovenous fistula; CS: corticosteroids, DVT: deep vein thrombosis; RA: radial artery; RCAVF: radiocephalic arteriovenous fistula.

a Possibly contributing to the observed complications.

b Patient had no complications at initial AVF ligation, but later had resection of an aneurysmatic dilatation of the perianastomotic area and the initial part of the fistula vein stump with secondary infection and several revisions. We believe the thrombosis of the perianastomotic segment of BA occurred at that time.

**Table 4:** Incidence of symptomatic arterial complications in the arm with a closed AVF in the full cohort—the retrospective part of the study (cases are presented in detail in Table 2); incidence of additional asymptomatic complications detected with prospective ultrasound examination in the prospective cohort (cases are presented in detail in Table 3); cumulative incidence of all (symptomatic and asymptomatic) complications in the full cohort.

Complications	Symptomatic complications	Additional asymptomatic complications on prospective ultrasound exams	All complications
Patients, *N*	60 (full cohort)	50 (prospective cohort)	60 (full cohort)
Aneurysms of BA, *n* (%)	3 (5)	3 (6)	6 (10)
Without complications With mural/occlusive thrombus	0 (0) 3 (5)	1 (2) 2 (4)	1 (2) 5 (8)
Significant arterial thrombosis, *n* (%)	5 (8)	7 (14)	12 (20)
From RA anastomosis to the elbow From RA/BA anastomosis up to mid-BA or AA From RA anastomosis to a BA aneurysm Perianastomotic segment of BA	1 (2) 4 (7) 0 (0) 0 (0)	3 (6) 2 (4) 1 (2) 1 (2)	4 (7) 6 (10) 1 (2) 1 (2)
Thrombosis of short perianastomotic segment of RA, *n* (%)	–	2 (4)	2 (3)
Kinking of BA, *n* (%)	1 (2)	9 (18)	10 (17)

BA: brachial artery; RA: radial artery; AA: axillary artery.

Patients could have more than one complication simultaneously.

The cumulative incidence of all complications (symptomatic and asymptomatic) is provided in Table 4. Altogether, in our national cohort of patients after AVF closure, 20% of patients developed significant arterial thrombosis and 10% developed brachial artery aneurysm, neither of which was associated with the site of anastomosis. AVF vintage was comparable in patients with and without arterial thrombosis [7 (IQR 4.75–12.5) versus 8 (IQR 5–11); *P* = .65] as well as with and without brachial artery aneurysm [10 (IQR 6.25–10) versus 8 (IQR 5–11); *P* = .78]. Some of the other potential factors influencing complications are analysed in Table 5 and none of them were statistically significant. Nevertheless, aneurysms were almost four times more common in men. Less significant complications included kinking of the brachial artery in 17% of patients, which was asymptomatic in the vast majority of cases. We cannot say whether it developed before or after AVF closure.

**Table 5:** Cumulative incidence (symptomatic and asymptomatic) of arterial complications in the arm with a closed AVF in the full cohort; comparison of some of potential factors influencing outcomes (comparison by Fisher exact test).

Potential factors	Significant arterial thrombosis	*P*-value	BA aneurysm	*P*-value
Male versus female	8/33 (24%) versus 4/27 (15%)	.52	5/33 (15%) versus 1/27 (4%)	.21
Arm versus forearm AVF	4/24 (17%) versus 8/36 (22%)	.75	2/24 (8%) versus 4/36 (11%)	1.00
Patients with versus without corticosteroids	8/37 (22%) versus 4/23 (17%)	.75	3/37 (8%) versus 3/23 (13%)	.67
Nephrologist versus vascular surgeon	7/28 (25%) versus 5/32 (16%)	.52	2/28 (7%) versus 4/32 (13%)	.68
Hypertension versus no hypertension	6/42 (14%) versus 6/18 (33%)	.16	4/42 (10%) versus 2/18 (11%)	1.00
Diabetes versus no diabetes	3/16 (19%) versus 9/44 (20%)	1.00	3/16 (19%) versus 3/44 (7%)	.33
ADPKD versus other primary kidney disease	3/9 (33%) versus 9/51 (18%)	.37	1/9 (11%) versus 5/51 (10%)	1.00

ADPKD: adult polycystic kidney disease.

Patients could have more than one complication simultaneously.

## DISCUSSION

To our knowledge, our study reports the largest cohort of patients with long-term follow-up after AVF closure, which allowed us to assess the cumulative incidence of significant arterial complications (thrombosis or aneurysm development) during a follow-up period of ≈10 years. The study showed that complications on the feeding artery after AVF closure were quite common and should therefore be taken into consideration when discussing AVF closure with patients.

It remains controversial whether a functional AVF should be electively closed in patients with a functioning kidney transplant. While most studies have focused on the effects of AVF closure on cardiac and kidney function [3–6], we conducted a study on complications in the arterial system of an arm with a closed AVF. We do not routinely close AVFs in our transplanted patients. Usually, the AVF is closed at the patient's request because of an aneurismal fistula vein, often associated with high flow, or other complications of the AVF. When considering closure of an AVF, the patient's kidney function and presence of acute or chronic graft rejection, but also the patient's cardiac function, should be evaluated. Perhaps most importantly, the possibility of creating a new AVF should be ensured, as most transplant patients will eventually return to haemodialysis. In addition to these well-established factors, complications in the arterial system after closure should also be considered, even in patients with a well-established indication for AVF closure.

The literature on complications after AVF closure is sparse. Development of a feeding artery aneurysm proximal to the anastomosis is a known phenomenon, although not mentioned in the guidelines [1]. A recent literature review collected 56 cases of brachial artery aneurysms published as case reports or case series and observed a male predominance [13], but the true incidence of complications cannot be estimated from case series. Moderately large cohorts of patients after AVF ligation either do not report long-term complications [19] or report no cases of arterial aneurysms [4, 20, 21], with the exception of aneurysms of the fistula vein stump after simple ligation [8]. There is one prospectively followed cohort of 29 transplant patients in which one patient developed a brachial artery aneurysm with a functioning AVF. Furthermore, one of nine patients with a ligated AVF also developed a brachial artery aneurysm (≈11% incidence) [11]. Our present study showed a similar incidence (10%) in a much larger sample (approximately half of aneurysms requiring surgical intervention). A large cross-sectional study by Janeckova *et al.* [18] found a 21% prevalence of brachial artery aneurysms (with a laxer definition of >1 cm) in 162 transplanted patients with either functional or non-functional AVFs. Eleven aneurysms were reported in 79 patients with non-functional (thrombosed or ligated) AVFs, giving a cumulative incidence of ≈14% [18], which is higher than in our study, but their definition was more lax.

The exact mechanism of feeding artery aneurysm development is not known. The formation of an arteriovenous anastomosis creates a low-resistance connection between the arterial and venous systems and significantly increases flow through the brachial artery [22, 23]. The increased shear stress on the arterial wall leads to vasodilatation, widening of the artery and loss of internal elastic lamina, ultimately leading to thinning of the arterial wall [23]. According to Laplace's law, a larger diameter of the artery increases the surface tension of the vessel wall, which further promotes the increase in diameter. When blood flow and vessel diameter reach a certain level, the process probably propagates itself further and may lead to a so-called megafistula [21]. This process is often more pronounced in transplant patients, as treatment with corticosteroids can further weaken the arterial wall and reduce its elasticity [24]. After AVF closure, the pressure within the arterial system abruptly increases again due to the increase in peripheral resistance, resulting in a further increase in wall tension, which may favour the formation of an aneurysm. This is likely the reason why most brachial artery aneurysms reported in the literature have occurred after AVF closure. In some cases, physical injury to the thin-walled artery could also be a contributing factor for aneurism formation, similar to cases of brachial artery aneurysms in patients without a prior AVF.

Feeding artery thrombosis is a rarely described phenomenon [6, 9]. Many of the moderately large cohorts report no such complications [4, 8, 19–21]. It is possible that some of the cases of thrombophlebitis of the fistula vein described in these reports [2, 4, 8] also included asymptomatic or oligosymptomatic thrombosis of the feeding artery, if an ultrasound exam was not performed. On the other hand, we found a very high cumulative incidence (20%) of significant arterial thrombosis. About half of patients were symptomatic, they developed thrombosis 1–7 years after AVF closure and in most cases it progressed to the brachial or even axillary artery. In addition, asymptomatic thrombosis was discovered in 14% of patients by prospective ultrasound exam. In more than half of the cases, the thrombosis was limited to the radial artery, but there were also cases in which the thrombosis spread more proximally.

The mechanisms of arterial thrombosis after AVF closure are likely multifactorial. Thrombosis that occurs several years after AVF closure likely has a secondary factor (minor injury, systemic inflammation/procoagulant state, etc.) that becomes significant, since the feeding artery is wide and thin-walled, with slow blood flow or even aneurysmatic. Indeed, all patients in our cohort with arterial thrombosis that did not remain limited to the radial artery but spread to the brachial artery had large brachial arteries (>10 mm) or a brachial artery aneurysm. The development of a thrombus in the stump of the ligated fistula vein and its propagation to the feeding artery may be another mechanism of thrombosis in the case of simple fistula vein ligation. We found a comparable incidence of complications in surgeries performed by nephrologists compared with vascular surgeons, which roughly corresponds to the comparison of simple vein ligation versus extirpation with artery reconstruction. Although ligation is a much simpler and shorter procedure [8], extirpation of the AVF with artery reconstruction should be preferred in AVFs with wide feeding arteries to prevent long-term complications. The asymptomatic thromboses likely occurred at the time of AVF closure and could be the result of spreading of thrombus from the stump of the fistula vein or could be induced by local thrombo-inflammation due to extensive tissue dissection combined with slow blood flow in an enlarged artery. None of the patients in our cohort with arterial thrombosis was on anticoagulant therapy at the time of AVF closure, but since in the entire cohort there were only two patients on anticoagulant therapy, this prevents a meaningful comparison. Nevertheless, to prevent these early thromboses, short-term (e.g. 1–3 months) perioperative prophylactic anticoagulation should be considered.

Although arterial thrombosis was asymptomatic in more than half of our cases, this is a serious complication. One should consider the patient's ability to perform physically demanding tasks and that significant arterial thrombosis precludes further AVF attempts on the affected limb, which usually significantly limits future options for vascular access. Flow reduction surgery (with aneurysm reduction when needed) should be considered as an alternative to AVF closure in selected patients with wide feeding arteries, to maintain appropriate blood flow and prevent thrombosis. Furthermore, regular screening for high AVF flow in physically active patients and flow reduction procedures prior to the development of a megafistula would not only protect the patient's cardiac function, but also reduce the possibility of arterial complications in case of spontaneous thrombosis or elective closure.

Our study has several limitations. We do not have data on clinical manifestations of atherosclerosis nor comprehensive ultrasound exams before AVF closure. We also do not have a control group of patients with a functional AVF after kidney transplantation, which would enable us to fully attribute the observed complications to the AVF closure.

To conclude, our study on a national cohort of transplant patients after AVF closure showed a rather high incidence of significant complications on the feeding artery (thrombosis or aneurysm development). These complications, sometimes developing several years after AVF closure, should be considered and presented to the patient when contemplating closure of a well-developed and long-standing AVF. We hypothesize that they develop because of a sudden haemodynamic change within a wide feeding artery with a thinned wall induced by AVF closure. Therefore, in many cases an AVF-preserving approach with flow reduction surgery might be preferred. Furthermore, if the AVF is closed, short-term perioperative prophylactic anticoagulation should be considered. Finally, examination of the entire feeding artery should be an integral part of the AVF ultrasound exam, especially in well-developed (high-flow or long-standing) AVFs, and follow-up exams should be considered after closure of a well-developed AVF.

## Data Availability

The raw data supporting the conclusions of this article will be made available by the authors upon request.
